# Knee extension strength and handgrip strength are important predictors of Timed Up and Go test performance among community-dwelling elderly women: a cross-sectional study

**DOI:** 10.1590/1516-3180.2020.0182.R1.30102020

**Published:** 2021-01-11

**Authors:** Diogo Carvalho Felício, José Elias, Bárbara Zille de Queiroz, Juliano Bergamaschine Mata Diz, Daniele Sirineu Pereira, Leani Souza Máximo Pereira

**Affiliations:** I PT, PhD. Physiotherapist, Postgraduate Program on Rehabilitation Sciences, Universidade Federal de Minas Gerais (UFMG), Belo Horizonte (MG), Brazil; Postgraduate Program on Rehabilitation Sciences and Functional-Physical Performance, School of Physiotherapy, Universidade Federal de Juiz de Fora (UFJF), Juiz de Fora (MG), Brazil.; II PT, MSc. Physiotherapist, Postgraduate Program on Rehabilitation Sciences and Functional-Physical Performance, School of Physiotherapy, Universidade Federal de Juiz de Fora (UFJF), Juiz de Fora (MG), Brazil.; III PT, PhD. Physiotherapist, Postgraduate Program on Rehabilitation Sciences, Universidade Federal de Minas Gerais (UFMG), Belo Horizonte (MG), Brazil.; IV PT, MSc. Physiotherapist, Postgraduate Program on Rehabilitation Sciences, Universidade Federal de Minas Gerais (UFMG), Belo Horizonte (MG), Brazil.; V PT, PhD. Physiotherapist, Postgraduate Program on Rehabilitation Sciences, Universidade Federal de Minas Gerais (UFMG), Belo Horizonte (MG), Brazil.; VI PT, PhD. Physiotherapist, Postgraduate Program on Rehabilitation Sciences, Universidade Federal de Minas Gerais (UFMG), Belo Horizonte (MG), Brazil.

**Keywords:** Aged, Gait, Locomotion, Muscle strength, Functionally-impaired elderly, Elderly women, Locomotor activity, Handgrip muscle strength

## Abstract

**BACKGROUND:**

Handgrip and knee extension strengths have each been used to characterize disability. However, it has been reported that the association between handgrip and knee extension strengths is weak.

**OBJECTIVE:**

To evaluate the influence of knee extensor and handgrip muscle strength on Timed Up and Go (TUG) test results among elderly women with worse (≥ 10 seconds) and better (< 10 seconds) performance, after controlling for confounders.

**DATA AND SETTING:**

Cross-sectional study on a sample selected according to convenience, carried out in a federal public institution of higher education.

**METHODS:**

Assessment of handgrip was carried out using the Jamar dynamometer (Lafayette Instrument Company, Inc., Lafayette, United States). Knee extensor muscle performance was measured using an isokinetic dynamometer (Biodex System 3 Pro; Biodex Medical Systems, Inc., United States), The confounding factors were education, age, comorbidities, body mass index and Geriatric Depression Scale and Human Activity Profile scores. Functional performance was assessed through the TUG test. A backward linear regression model was used.

**RESULTS:**

127 elderly women performed the TUG test in more than 10 seconds and 93 in less than 10 seconds. However, regardless of test performance, handgrip strength and knee extension strength comprised the reduced final model.

**CONCLUSIONS:**

Knee extension strength and handgrip strength might be particularly useful indicators for measuring disability.

## INTRODUCTION

Knee extension and handgrip strengths have each been used to predict functional performance.^1^ Caballer et al. observed significant correlations (-0.57) between rectus femoral cross-sectional area and Timed Up and Go (TUG) test performance in a sample of 122 adults aged 65 and older.^2^ Di Monaco et al. evaluated 123 elderly women and found a significant correlation (-0.41) between handgrip strength and time taken to complete the TUG test.^3^

However, it has been reported that the association between handgrip strength and knee extension strength is weak. Felicio et al. investigated the correlation between handgrip strength and the isokinetic muscle performance of knee extensors and flexors among elderly women living in the community (n = 221). Most of the muscle variables analyzed with their isokinetic dynamometer did not show any significant correlation with handgrip strength.^4^ In a study on 764 elderly individuals, Chan et al. found a weak association between knee extension strength and handgrip muscle strength when the results were adjusted for sex and age (R^2^ = 0.17).^5^

## OBJECTIVE

To evaluate the influence of knee extensor strength and handgrip muscle strength in the TUG test among elderly women with worse (≥ 10 seconds) and better (<10 seconds) performance, after controlling for confounders.

## METHODS

### Participants and setting

This was a cross-sectional study approved by a local ethics committee on May 31, 2010 (ETIC 0038.0.203.000-10). Sample selection was carried out according to convenience. The study included women living in the community aged 65 years and over. The exclusion criteria were cognitive dysfunction,^6^ acute inflammatory conditions, musculoskeletal discomfort and visual or hearing losses.

### Testing procedures

#### Muscle performance of knee extensors

The muscle performance of the knee extensors was measured using the Biodex System 3 Pro isokinetic dynamometer (Biodex Medical System, Shirley, United States). The data analysis was carried out using only the results obtained from the dominant lower limb, and concentric-concentric mode was selected for the assessment.^7^ The angular velocity selected was 60°/switch, with five repetitions, and the isokinetic variable used was work/body mass because this is most representative of muscle function.^8^

#### Handgrip strength

Handgrip strength was measured by means of a maximal isometric test, using the PC5030JI Jamar device (Sammons Preston, Bolingbrook, United States), performed on the dominant upper limb. The participants were positioned in accordance with the recommendations of the American Society of Hand Therapy. An average of three trials was calculated to obtain a final score.^9^

#### Confounding factors

The confounding factors included in the analysis were education, age, number of comorbidities, body mass index (BMI), Geriatric Depression Scale (GDS-15) score^10^ and Human Activity Profile (HAP) score.^11^

#### Timed Up and Go test

The Timed Up and Go (TUG) test involves getting up from a chair, walking three meters around a marker placed on the floor, coming back to the same position and sitting down on the chair again.^12^

#### Data analysis

Comparisons between the groups were made using the t test. A backward linear regression model was used to investigate the influence of knee extensor strength and handgrip muscle strength on the TUG test results among elderly women with worse (≥ 10 seconds) and better (< 10 seconds) performance. The dependent variable was the TUG test result. The independent variables were age, education, BMI, HAP, GDS, mini-mental state examination (MMSE), number of comorbidities, knee extensor strength and handgrip muscle strength. Regarding the model assumptions, multicollinearity was considered to have an inflation factor of variance of > 10. The analysis on homoscedasticity was performed using graphical observations of predicted and observed values. The independence of residuals was determined using the Durbin-Watson test. For all analyses, we used a significance level of 0.05. The statistical analyses were carried out using the Statistical Package for the Social Sciences (PASW Data Collection, version 20.0; SPSS, Chicago, United States).

## RESULTS

A total of 220 elderly women met the inclusion criteria for the present study. The descriptive sample characteristics are shown in Table 1.

**Table 1.** Descriptive sample characteristics (n = 220)

Variables	TUG > 10 s (n = 127) Mean ± standard deviation	TUG < 10 s (n = 93) Mean ± standard deviation	P-value
Age, years	71.2 ± 4.8	70.6 ± 5.0	0.43
Education, years	5.8 ± 4.2	6.2 ± 3.9	0.40
Body mass index, kg/m^2^	29.8 ± 5.8	28.3 ± 4.5	0.02^*^
Human activity profile, 0-94	69.0 ± 11.9	75.0 ± 9.7	< 0.001^*^
Geriatric Depression Scale, 0-15	3.7 ± 2.9	3.2 ± 2.5	0.23
Mini-mental state examination, 0-30	25.6 ± 3.0	26.3 ± 2.9	0.10
Comorbidities, n	2.7 ± 1.5	2.4 ± 1.5	0.16
Work/body weight of knee extensors, %	115.1 ± 29.4	127.8 ± 34.1	< 0.001^*^
Handgrip strength, kgf	20.4 ± 4.7	22.0 ± 4.2	0.01^*^
Timed Up and Go test, seconds	11.7 ± 1.6	8.7 ± 1.0	0.00^*^

^*^Statistically significant.

Table 2 presents the results from the reduced backward linear regression model.

**Table 2.** Results from linear regression model (n = 220)

Dependent variable = TUG ≥ 10 s (n = 127)		
**Model**	**Adjusted R^2^**	**P-value**
**1**	**0.20**	< 0.001^*^
Model 1: education (standardized β = -0.17); human activity profile (standardized β = -0.29); work/body weight of knee extensors (standardized β = -0.07); handgrip strength (standardized β = -0.23)		
Dependent variable = TUG < 10 s (n = 93)		
**Model**	**Adjusted R^2^**	**P-value**
**2**	**0.06**	0.03^*^
Model 2: age (standardized β = -0.26); work/body weight of knee extensors (standardized β = -0.12); handgrip strength (standardized β = -0.09)		

^*^Statistically significant.

## DISCUSSION

A single measurement to provide an overall estimate of functionality reduces the time and cost of evaluation. The present study presents preliminary evidence of the importance of knee extension strength and handgrip strength in relation to functional performance, even after adjustment.

Handgrip strength is a measurement that has been widely studied in the literature and can be used as a screening tool for impairments among elderly people. However, this measurement does not eliminate the need for specific assessment. In this regard, Bohannon^13^ compared the capacities of handgrip strength and knee extension strength to correlate with gait speed among elderly people. Knee extension forces showed a correlation with gait speed, whereas grip strength forces did not. Knee extension force measurements satisfactorily identified patients with gait speeds < 0.40 m/s. These results demonstrated the importance of lower limb strength over handgrip strength, in relation to functional performance.

Clinicians need to be cautious in generalizing from handgrip strength as a predictor of functional status. Muscles evaluated using a manual dynamometer are not recruited in tasks that involve supporting bodyweight; static contractions are rarely required for daily activities and the muscle strength of the upper and lower limbs is subject to differential decline.^14^ Furthermore, other factors may have an inﬂuence on handgrip strength. These include grip size,^15^ dominance,^16^ genetic factors and anthropometric variables.^17^^,^^18^

Martien et al.^19^ investigated whether knee extension strength is a better predictor of functional performance than handgrip strength. Their study sample consisted of 770 elderly people. Strength was measured using handgrip and knee extension tests. Functional performance was assessed by means of the six-minute walking distance test and a modified physical performance test. They found that only knee extension strength was clearly more predictive than handgrip strength. Recently, Wiśniowska-Szurlej et al. showed that there was a negative correlation between TUG test results and lower-limb strength.^20^ This was supported by the findings of Zarzeczny et al.,^21^ who showed that the results from the 30-second chair stand test were negatively correlated with those from the TUG test. Whenever possible, handgrip strength and knee extension strength should be used together to assess muscle strength and identify individuals who are vulnerable to poor health outcomes.

## CONCLUSIONS

Knee extension strength and handgrip strength might be particularly useful indicators for screening for functional performance among elderly people. Whenever possible, the assessment strategies should be used in a complementary manner.
